# Could pulmonary low-dose radiation therapy be an alternative treatment for patients with COVID-19 pneumonia? Preliminary results of a multicenter SEOR-GICOR nonrandomized prospective trial (IPACOVID trial)

**DOI:** 10.1007/s00066-021-01803-3

**Published:** 2021-07-06

**Authors:** M. Arenas, M. Algara, G. De Febrer, C. Rubio, X. Sanz, M. A. de la Casa, C. Vasco, J. Marín, P. Fernández-Letón, J. Villar, L. Torres-Royo, P. Villares, I. Membrive, J. Acosta, M. López-Cano, P. Araguas, J. Quera, F. Rodríguez-Tomás, A. Montero

**Affiliations:** 1 Universitat Rovira i Virgili, Tarragona, Spain; 2 Institut d’Investigacions Pere Virgili, Tarragona, Spain; 3 Department of Radiation Oncology, Hospital Universitari Sant Joan de Reus, Tarragona, Spain; 4 Department of Radiation Oncology, Hospital del Mar, Barcelona, Spain; 5 Autonomous University of Barcelona, Barcelona, Spain; 6 Institut Hospital del Mar d’Investigacions Mèdiques, Barcelona, Spain; 7 Department of Geriatric and Palliative care, Hospital Universitari Sant Joan de Reus, Tarragona, Spain; 8 Department of Radiation Oncology, HM Hospitales., Madrid, Spain; 9 Pompeu Fabra University Barcelona, Barcelona, Spain; 10 Department of Critical Care, Hospital del Mar, Barcelona, Spain; 11 Department of Medical Physics, HM Hospitales, Madrid, Spain; 12 Department of Infection Diseases, Hospital del Mar, Barcelona, Spain; 13 Department of Internal Medicine, HM Hospitales, Madrid, Spain

**Keywords:** COVID-19 pneumonia, Low-dose radiation therapy, Treatment outcome, Anti-inflammatory effects, Lung irradiation

## Abstract

**Purpose:**

To evaluate the efficacy and safety of lung low-dose radiation therapy (LD-RT) for pneumonia in patients with coronavirus disease 2019 (COVID-19).

**Materials and methods:**

Inclusion criteria comprised patients with COVID-19-related moderate–severe pneumonia warranting hospitalization with supplemental O_2_ and not candidates for admission to the intensive care unit because of comorbidities or general status. All patients received single lung dose of 0.5 Gy. Respiratory and systemic inflammatory parameters were evaluated before irradiation, at 24 h and 1 week after LD-RT. Primary endpoint was increased in the ratio of arterial oxygen partial pressure (PaO_2_) or the pulse oximetry saturation (SpO_2_) to fractional inspired oxygen (FiO_2_) ratio of at least 20% at 24 h with respect to the preirradiation value.

**Results:**

Between June and November 2020, 36 patients with COVID-19 pneumonia and a mean age of 84 years were enrolled. Seventeen were women and 19 were men and all of them had comorbidities. All patients had bilateral pulmonary infiltrates on chest X‑ray. All patients received dexamethasone treatment. Mean SpO_2_ pretreatment value was 94.28% and the SpO_2_/FiO_2_ ratio varied from 255 mm Hg to 283 mm Hg at 24 h and to 381 mm Hg at 1 week, respectively. In those who survived (23/36, 64%), a significant improvement was observed in the percentage of lung involvement in the CT scan at 1 week after LD-RT. No adverse effects related to radiation treatment have been reported.

**Conclusions:**

LD-RT appears to be a feasible and safe option in a population with COVID-19 bilateral interstitial pneumonia in the presence of significant comorbidities.

**Supplementary Information:**

The online version of this article (10.1007/s00066-021-01803-3) contains supplementary material, which is available to authorized users.

## Introduction

Since the beginning of 2020 when coronavirus disease 2019 (COVID-19) appeared, the world has been battling against a new and practically unknown highly contagious viral infection which yet has no cure. Although the majority of infected people develop only mild, if not practically asymptomatic, clinical effects, most serious clinical manifestations of the severe acute respiratory syndrome (SARS)-CoV‑2 virus infection derive mainly from the hyperinflammatory lung response that some infected patients experience, which leads to SARS, the cause of death from COVID-19 in many cases [1].

Numerous pharmacological alternatives have been proposed although none have proven to be definitively effective. In this context, a number of groups in different parts of the world have been exploring the idea of a one-off effective radiotherapy treatments in lung pneumonia. Several publications have proposed theoretical bases for the usefulness of radiotherapy in COVID-19 and although the idea of using radiation therapy to treat different respiratory disorders is not new [2–4], it has generally been met with prejudice and fear that often accompany the therapeutic use of ionizing radiation. Advances in radiobiology help us to understand the efficacy of low-dose radiation therapy (LD-RT) as a plausible anti-inflammatory treatment. Radiotherapy doses conventionally used in the treatment of neoplastic diseases induce the production and release of pro-inflammatory cytokines. However, low doses of between 0.3–0.7 Gy induce a totally opposite phenotype thanks to their effect on leukocytes, macrophages, polymorphonuclear cells, and vascular endothelial cells. LD-RT provokes a decrease in pro-inflammatory mediators, such as reactive oxygen species (ROS), nitric oxide synthetase (iNOS), tumor necrosis factor (TNF)-α, selectin L‑ and E‑, or interleukin (IL)-beta 1. Additionally, LD-RT favors a change in macrophage polarization from a pro-inflammatory M‑1 phenotype to an anti-inflammatory M‑2 phenotype at the same time as increasing the secretion of anti-inflammatory mediator transforming anti-inflammatory cytokine growth factor β1 (TGF-β1) and apoptosis mediators nuclear factor kappa-beta (NF-κB) [5–9]. Based on this knowledge, several studies of pulmonary LD-RT in COVID-19 pneumonia have been initiated in different countries [10].

We hypothesized that pulmonary LD-RT can prevent or reduce the lung inflammatory cascade produced by COVID-19 and it can be an alternative treatment for these patients. We report here the results of a whole lung LD-RT multicenter trial on 36 patients with COVID-19-related pneumonia.

## Materials and methods

The protocol details of this multicenter, nonrandomized prospective trial have been published previously [11]. The main study objective was to evaluate the efficacy of single pulmonary LD-RT (0.5 Gy), evaluated according to an increase in arterial oxygen partial pressure (PaO_2_) to fractional inspired oxygen (FiO_2_) (PaO_2_/FiO_2_) ratio or the pulse oximetry saturation (SpO_2_) to FiO_2_ (SpO_2_/FiO_2_) ratio of at least 20% at 24 h with respect to the pre-irradiation value in at least 30% of the treated and evaluable patients. Secondary objectives included positive changes in radiological image, mortality at 15 and 30 days after LD-RT, and the effects on inflammatory blood parameters including the determination of C‑reactive protein (CRP), interleukin‑6 (IL-6), ferritin, D‑dimer (DD) and lactic acid dehydrogenase (LDH). We consider necessary to note that although this trial was designed in 2 phases: a first exploratory phase that intended to recruit 10 patients to evaluate the viability and efficacy of single-fraction pulmonary RT-LD, and a second, nonrandomized comparative phase with a control group, its interest during the COVID-19 pandemic triggered recruitment from the initial phase to the 36 patients we now report.

Inclusion criteria comprised patients with COVID-19-related moderate–severe pneumonia who were not candidates for admission to the intensive care unit (ICU) because of comorbidities or low performance status. The LD-RT plan comprised a single dose of 0.5 Gy, although the protocol included the possibility of administering a second dose of 0.5 Gy after 48 h, depending on the response to the first and individualizing every case. A planning computed tomography (CT) scan with axial images obtained at 3 mm intervals throughout the lung was acquired from all patients who were candidates for LD-RT. Clinical target volume (CTV) included both lungs. The radiotherapy plan consisted of anterior posterior–posterior anterior 2‑field conformal treatment for both lungs without any type of protection (Supplementary information Figure A1). Due to the ultra-low prescribed, defining dose, specific constraints were not considered necessary for healthy organs beyond the ALARA (as low as reasonably achievable) principle.

To evaluate the efficacy of the treatment, variations in the daily oxygen supply needs according to the FiO_2_, which represents the percentage of oxygen participating in gas exchange, and periodic determinations of PaO_2_/FiO_2_ or SpO_2_/FiO_2_ ratio, radiologic evaluation of lung infiltrates and determination of inflammation markers were carried out and measured before irradiation (basal) and at 24 h and at 1 week after treatment. In some cases (13/36), it was also possible to analyze all parameters at 1 month after LD-RT.

To assess the functional status and potential risk of the study population, we decided to use two existing tools designed for this purpose. The Barthel Index was used to evaluate patients’ functional independence by means of an ordinal scale used to measure performance in activities of daily living (ADL), and the CURB-65 Severity Score is a clinical prediction rule that has been validated for estimating mortality of community-acquired pneumonia measuring Confusion, blood Urea nitrogen (BUN), Respiratory rate, Blood pressure and age over 65. Due to the cognitive impairment and several comorbidities that all elderly patients presented in this trial, we classified the groups into three different subgroups: survivors (group A), deaths from COVID-19 (group B) and deaths from other causes (group C), in order to support the initial supposition of severe cognitive impairment and associated increased risk of death.

All statistical analysis and graph representations were performed by SPSS software (SPSS 22.0, Chicago, IL, USA), ‘R’ software version 4.0.2 (https://www.r-project.org/) and GraphPad Prism 6.01 (GraphPad Software, San Diego, CA, USA). To assess the distribution of the variables was used the Kolmogorov–Smirnov test. Through the Mann–Whitney *U*-test (nonparametric) we evaluated differences between any two groups of variables and for comparisons of dependent variables we used the Wilcoxon signed-rank test. To assess differences between qualitative variables we employed the χ^2^ test. Medians and interquartile ranges were expressed for quantitative variables and frequencies and percentages for qualitative variables. We considered significant differences when the *p*-value was < 0.05.

This protocol conforms to international regulations and is in accordance with the recommendations established in the Declaration of Helsinki. The study was approved by the Institution Research Board of each center and is registered in ClinicalTrials.gov (NCT NCT04380818).

## Results

Between June and November 2020, 36 patients with COVID-19 pneumonia participated in this trial. All of them signed the consent form before treatment. Table 1 gives the patients’ baseline characteristics. In all, 19 men and 17 women were included, with a mean age of 84 years, all of them presenting multiple comorbidities, the most common being cardiovascular disease. Patients were classified according to the Barthel Index as ‘independent’ in 6 cases (16.7%), ‘mild dependence’ in 12 (33.3%), ‘moderate dependence’ in 7 (19.4%), ‘severe dependence’ in 8 (22.2%) and ‘completely dependent’ in 3 (8.3%). All patients had bilateral pulmonary infiltrates on chest X‑ray at diagnosis. The radiological pneumonia, measured according to percentage of lung parenchyma affected on pretreatment CT, showed that 27.8% presented affection of > 75%, 50% of 51–75%, 19.4% of 26–50% and 2.8% only 5–25%. A mean pre-irradiation SpO_2_ of 94.28%. Mean value of CRP was 9 mg/dL (1.14–24.7 mg/dL). All patients received dexamethasone treatment, one patient also received tocilizumab, one received remdesivir, and a third patient received both of them. All patients received a dose of 0.5 Gy and although considered in trial protocol, a second 0.5 Gy dose was not administered in any patient. During the follow-up month, no patients presented radiation-related adverse effects.

**Table 1:** Clinical characteristics of patients with COVID-19 treated with low-dose radiation therapy

**Variables**	**(*n*** **=** **36)**
*Age*	83.64 (8.11)^b^
*Sex*	
Female	17 (47.2)^a^
Male	19 (52.8)
*Neurologic diseases*	10 (27.8)^b^
*Cardiovascular diseases*	30 (83.3)^b^
*Respiratory diseases*	12 (33.3)^b^
*Other comorbidities*	31 (86.1)^b^
*Days with symptoms*	5.72 (1.54)^b^
*Functional status (Barthel Index)*	
Independent	6 (16.7)^a^
Minimally dependent	12 (33.3)
Partially dependent	7 (19.4)
Very dependent	8 (22.2)
Total dependent	3 (8.3)
*Geriatric Depression Scale (GDS)*	
No cognitive decline	18 (50)^a^
Very mild cognitive decline	8 (22.2)
Mild cognitive decline	5 (13.9)
Moderate cognitive decline	–
Moderately severe cognitive decline	2 (5.6)
Severe cognitive decline	3 (8.3)
Very severe cognitive decline	–
*Pharmacological treatment*	
Corticosteroids (dexamethasone)	36 (100)^a^
Remdesivir	2 (5.6)
Tocelizumab	2 (5.6)
Basal SpO_2_	94.28 (2.85)^b^
Basal SaFi	255.42 (117.75)^b^
Mild	18 (50)^a^
Moderate	5 (13.9)
Severe	13 (36.1)
Basal PaFi	251.39 (128.19)^b^
Mild	25 (69.4)^a^
Moderate	4 (11.1)
Severe	7 (19.4)
*CURB-65 Score*	
1 points	–
2 points	10 (27.8)^a^
3 points	17 (47.2)
4 points	9 (25)
*CT lung involvement (%)*	
< 5%	–
5–25%	1 (2.8)^a^
26–50%	7 (19.4)
51–75%	18 (50)
> 75%	10 (27.8)
*Final status*	
Alive	23 (63.9)^a^
Death due to COVID-19	8 (22.2)
Death due to other causes	5 (13.9)

*CT* Computed Tomography; *CURB-65* validated clinical prediction score for predicting mortality in community-acquired pneumonia and infection of any site including measurement of: Confusion of new onset, Blood Urea Nitrogen, Respiratory rate, Blood pressure and Age; *PaFi* ratio of arterial oxygen partial pressure (PaO_2_) to Fractional Inspired Oxygen (FiO_2_); *SpO*_*2*_ oxygen saturation; *SaFi* ratio of SpO_2_ to FiO_2_

^a^Results shown as frequencies and percentages in parenthesis

^b^Results shown as means and standard deviations in parenthesis

**Fig. 1:**
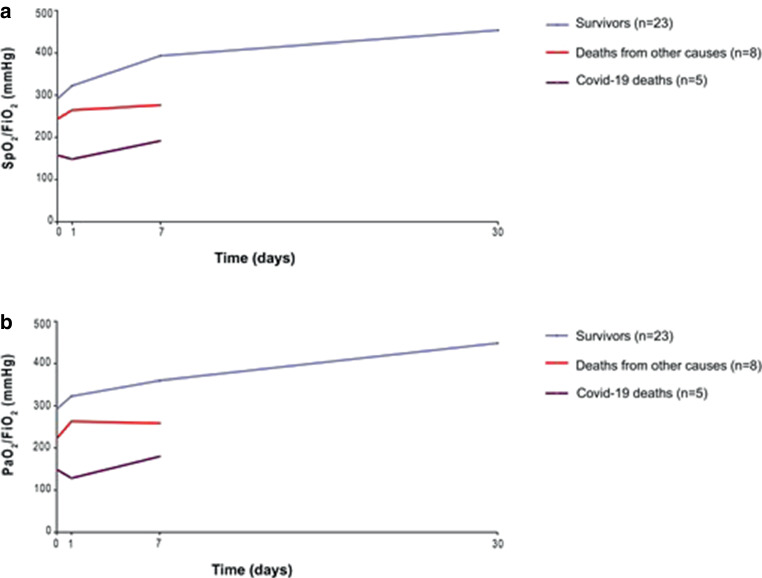
Evolution of respiratory parameters (SpO_2_/FiO_2_ [**a**] and PaO_2_/FiO_2_ [**b**]) in all patients with COVID-19 treated with low-dose radiation therapy classified by survivors, COVID-19 deaths and deaths from other causes

**Fig. 2:**
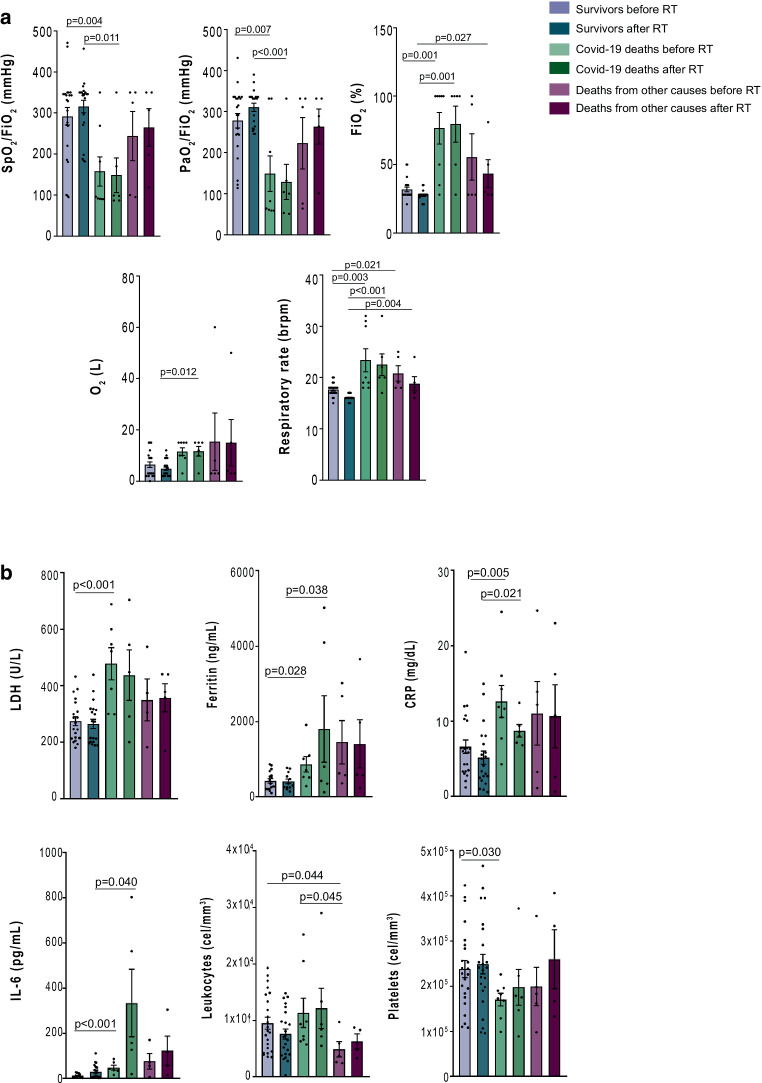
Selected respiratory (**a**) and biochemical (**b**) parameters comparisons between survivors, COVID-19 deaths and deaths from other causes before treatment and at 24 h after received treatment with low-dose radiation therapy (RT)

The evaluation of respiratory parameters at 24 h was possible for 34/36 patients (94.4%). SpO_2_/FiO_2_ and PaO_2_/FiO_2_ values were significantly higher (*p* < 0.01) in survivors than COVID-19 deaths only at baseline and 24 h after LD-RT. Seventeen patients (50%) presented an improvement of SpO_2_/FiO_2_, with a mean percentage of 38.82% compared to baseline value. At 1 week, 25/36 were evaluable and of them, 21 patients (84%) presented a mean SpO_2_/FiO_2_ improvement of 76%, while the other 4 patients did not present any improvement. We were able to examine the first 13 patients after 1 month of LD-RT at the time of the statistical evaluation, observing that none of them needed any supplemental oxygen therapy, and SpO_2_/FiO_2_ ratio was higher in all of them, increasing its value with a mean of 77.98% (463 mm Hg) (Fig. 1). Fig. 2 shows all respiratory parameters (2A) and inflammatory biochemical results (2B) before and at 24 h after LD-RT in all three groups. In groups A and C, there was an improvement in the respiratory parameters (SpO_2_/FiO_2_, PaO_2_/FiO_2_ and FiO_2_). In all groups a decrease in CRP was observed. Supplementary Information Figure A2 shows a significant improvement in the respiratory parameters and biochemical results before and after LD-RT. Table 2 shows biochemical variables of patients with COVID-19, classified by survivors, COVID-19 deaths and deaths from other causes, before, at 24 h, 1 week, and 1 month after LD-RT.

**Table 2:** Biochemical variables of patients with COVID-19, classified by survivors, COVID-19 deaths and deaths from other causes, before, at 24 h, 1 week and 1 month after low-dose radiation therapy (LD-RT)

	Survivors (*n* = 23)				COVID-19 deaths (*n* = 8)		Deaths from other causes (*n* = 5)	
	Before	24 h after	1 week after	1 month after	Before	24 h after	Before	24 h after
CD4	286.5 (144.5–377.5)	383 (211.5–566.5)	478 (230–824)^B,D^	515 (406.5–749)^A,D^	273.5 (187.7–318.7)	257 (234–493)	355.5 (205.2–493.7)	282.5 (244–500.2)
CD8	112 (78.7–223.2)	129 (50.5–256)^A^	181 (110.5–310.5)^A^	406.5 (204.5–640.7)^B,E,G^	169.5 (54.5–350.5)	193.5 (89.7–272.5)	133 (106–213.2)	180.5 (88–204.7)
CRP	6 (3.4–10.3)	4.6 (2.4–8.1)^A^	1.4 (0.3–5.8)^C,E^	0.7 (0.3–1.4)^C,F,G^	11.8 (8.4–15.6)^b^	8.3 (7.1–10.1)^d^	12.2 (2.2–19.3)	10.8 (1.6–19.6)
D‑Dimer	1000 (640–1813)	920 (680–2070)	995 (637.5–1990)	1490 (700–3100)	1570 (644.7–3037.5)	1595 (1057.5–3357.5)	770 (559.5–4590)	1304 (907.5–4889.5)
Ferritin	535.9 (247–937)	606 (256–1639.5)	493 (225.2–868.2)	265 (57–650.5)^A,D^	861 (522.1–1708.5)^a^	614 (296.8–4327.7)^d^	628 (471–2849)	591 (411–2816)
IL‑6	12 (7.7–26.7)	18 (6.7–57)	12.2 (5.0–26.2)^D^	11.5 (8–22)^D^	44 (35.5–87)^c^	158 (74.1–683.5)^d,e^	64.5 (19.2–146.5)	87 (26–258.2)
LDH	277.5 (214.5–344.2)	273 (201–317.5)	218.5 (170.7–284.5)^C,F^	201 (183.2–226.5)^A,E^	522 (300–600)^c^	448 (245.5–624.5)	339.5 (212.7–497)	378 (263.5–440.5)
Leukocytes	8140 (4520–14600)	7070 (3990–11460)^B^	7995 (5535–11530)	5210 (4350–7075)^A,G^	8280 (6392.5–17832.5)	8880 (6762.5–17267.5)	3790 (2535–7840)^a,e^	6565 (3675–8637.5)
Lymphocytes	790 (570–1370)	920 (590–1410)	1023 (525–1812.5)	1401 (1025–1612)^B,D^	750 (442.5–1113.5)	840 (558.2–5872.5)	530 (345–995)	825 (442.5–960)
Platelets	228000 (175000–291000)	241000 (190000–327000)	242500 (135000–337500)	215000 (116500–304250)	177500 (138750–194500)^a^	177000 (132500–260250)	183000 (130000–276500)	250000 (141500–387750)

Values are shown as medians (interquartile ranges). A = *p* < 0.05; B = *p* < 0.01, C = *p* < 0.001 with respect to before RT survivor group; D = *p* < 0.05, E = *p* < 0.01, F = *p* < 0.001 with respect to 24 h after RT survivor group; G = *p* < 0.05, with respect to 1 week after RT survivor group; a = *p* < 0.05, b = *p* < 0.01, c = *p* < 0.001, with respect to before RT survivor group; d = *p* < 0.05, with respect to 24h after RT survivor group; e = *p* < 0.05, with respect to before RT Covid-19 death group. *CD4* cluster of quadruple differentiation; *CD8* cluster of differentiation 8; *CRP* C-reactive protein; *IL‑6* interleukin 6; *LDH* lactate dehydrogenase. CD4, CD8, leukocytes, lymphocytes and platelets are expressed as cells/mm^3^; CRP as mg/dL, D‑Dimer as mcg/L, Ferritin as ng/mL, IL‑6 as pg/mL and LDH as U/L

Causes of patients’ death are shown in Table 3. Seven patients (19.4%) died from COVID-19 in the first week and 1 patient died on day 11 following LD-RT. All of them presented a high CURB-65 value, 3 and 4 points, and bilateral pneumonia with > 50% lung affection on CT. Additionally, 5 patients (13.8%) died from other causes during the follow-up. Supplementary Information Table A1 shows the evaluation of CURB-65 in survivors, COVID-19 deaths and deaths from other causes evaluated before LD-RT, at 24 h, at 1 week and at 1 month after LD-RT. Those patients who died of COVID-19 pneumonia had a significantly worse CURB-65 score before and at 24 h after LD-RT, confirming that high-risk pneumonia is a predictor of death (*p* < 0.01). Supplementary Information Figure A3 shows an improvement in the evolution of the radiological manifestations of COVID-19 pneumonia in one patient. No statistically significant differences were found in the 3 subgroups according to the Barthel Index, probably because of the small sample size in some subgroups, although there seems to be a trend towards a greater degree of dependence in group B (Supplementary Information Table A2).

**Table 3:** Causes of death, from COVID-19 or other causes following low-dose radiation therapy (LD-RT)

Patient	Death day after LD-RT	COVID-19	Age	Reason
1	1^st^	Yes	91	Severe cognitive impairment and constantly removed the ventilation mask herself
2	2^nd^	Yes	86	Acute respiratory distress secondary to COVID-19 (SaFi had worsened by 3%)
3	3^rd^	Yes	72	Severe cognitive impairment and constantly removed the ventilation mask herself
4	3^rd^	Yes	86	Acute respiratory distress secondary to COVID-19 (SaFi had improved a 3%)
5	4^th^	Yes	89	Severe cognitive impairment and constantly removed the ventilation mask herself
6	6^th^	Yes	88	Acute respiratory distress secondary to COVID-19
7	7^th^	Yes	88	Acute respiratory distress secondary to COVID-19 (SaFi had improved by 7%)
8	7^th^	No	74	Septic infection of unknown origin without response to empiric antibiotic administration
9	8^th^	No	73	Bronchoaspiration, having a subdural hemorrhage recent intervention. SaFi was stable without changes since basal determination (350 mm Hg)
10	11^th^	Yes	87	Acute respiratory distress secondary to COVID-19 (SaFi had worsened by 45% in 7 days)
11	12^th^	No	60	Severe worsening of his chronic renal failure under hemodialysis treatment
12	14^th^	No	55	Esophageal varices hemorrhage, having a known antecedent of enolic hepatic disease (he had improved SaFi from basal 95 mm Hg to 277 mm Hg in one week)
13	25^th^	No	85	Severe pulmonary thromboembolism

## Discussion

When SARS-CoV‑2 infects the lungs of a previously healthy patient by binding to the membrane receptor for angiotensin converting enzyme‑2 (ACE2), it stimulates a series of intracellular pathways favoring the release of pro-inflammatory cytokines and the recruitment of immune cells resulting in the induction of a hyperinflammatory state. That appears to be the key triggering mechanism for the most severe forms of infection [12]. To date, the only measures that have shown any degree of efficacy are those aimed at combating the process of inflammation and pulmonary SARS [13]. The treatments specifically directed against the overexpression of known mediators of inflammation such as IL‑1 or IL‑6 are among the few measures that have shown certain efficacy [14, 15]. The RECOVERY Collaborative Group randomized phase III study showed that the use of dexamethasone reduced death among those who were receiving either invasive mechanical ventilation or oxygen alone and reinforced the usefulness of corticosteroids for COVID-19 patients [16]. In the double-blind, randomized Adaptive COVID-19 Treatment Trial (ACTT-1), the antiviral agent remdesivir has been shown to be more effective than a placebo in hastening the recovery of hospitalized patients with COVID-19 pneumonia [17]. Dexamethasone and remdesivir are the only treatments that have shown true efficacy against COVID-19 [18]. Other strategies, such as the use of plasma from recovered convalescent patients might reduce the risk of death and could be an attractive strategy if subsequent studies confirm its efficacy [19].

Due to the lack of a definitive treatment and in accordance with the previously mentioned theoretical and experimental bases, the efficacy of LD-RT has been hypothesized for the treatment of respiratory complications associated with COVID-19 and numerous studies have been initiated by various groups [10]. The idea of using LD-RT to the lungs to treat pneumonia is not new. The review by Calabrese et al. collected evidence of 863 cases of pneumonia treated with LD-RT between 1905 and 1946 [4]. Observed results suggested the efficacy of LD-RT, although it must be kept in mind that these are old studies of retrospective nature, methodologically debatable according to present standards, using various treatment techniques considered obsolete today and many of them lacking a control group. Although when it was published it showed consistent data with the normal evolution of pneumonia at that time, it would be advisable to handle those results with caution. The mechanism of LD-RT, such as those proposed for the treatment of COVID-19 pneumonia, has different actions, including the reduction of pro-inflammatory cytokines while inhibiting the interaction between polymorphonuclear leukocytes and the vascular endothelium, and favors the polarization of lung macrophages from a pro-inflammatory M‑1 phenotype to an anti-inflammatory M‑2 phenotype [20, 21]. These changes, which favor the establishment of a local anti-inflammatory environment, would explain the clinical effects of LD-RT. Studies of pulmonary LD-RT currently underway are focusing on patients with presence of confirmed COVID-19 disease and radiologically evident pneumonia. Most trials have administered a single fraction of 0.5–1.5 Gy to both lungs, although three studies have considered administering a second identical fraction if there is no adequate response after the first during a time interval that varies between 24 and 240 h after the first fraction. The main objective of many of them is an improvement in oxygen saturation rates, although some studies also evaluated other aspects, such as the length of hospital stay, the need for admission to ICU, an improvement in radiology or the associated crude mortality rates [22]. In our trial, the primary endpoint of improving the PaO_2_/FiO_2_ or SpO_2_/FiO_2_ in 36 consecutive treated patients has been reached without any observed harmful effects attributable to the treatment.

To date, five investigation groups have published results from the use of LD-RT for patients with SARS-CoV‑2 pneumonia. One trial has presented preliminary results for 2 patients and one case report has also been published. Ameri et al. have presented the results of an Iranian study (NCT04390412) on 5 patients who received a single dose of 0.5 Gy to both lungs. In 4/5 patients, an improvement in clinical parameters (blood oxygenation and body temperature) and inflammatory markers (IL‑6 and CRP levels) was observed on the first day after treatment. No patient received any other specific treatment for COVID-19 infection. One patient died after 3 days, 1 patient chose to drop out of the study after 3 days, and another 3 patients were discharged. No complications related to LD-RT were reported [23]. Preliminary results of the Emory University Hospital RESCUE 1–19 trial have been published by Hess et al. Five patients diagnosed with COVID-19 pneumonia received a single 1.5 Gy fraction over both lungs. Four of the 5 patients experienced clinical recovery, 3 of them within the first 24 h after irradiation and could be discharged after a median admission of 12 days. No patient received treatment with drugs directed against COVID-19 in the days before or after lung LD-RT. The authors report no acute toxicity attributable to treatment [24]. These same authors recently updated their results during the American Society for Radiation Oncology (ASTRO) 2020 Annual Meeting. The results have been presented for 20 patients, 10 of whom received low-dose bilateral pulmonary irradiation while another 10 served as controls. They reported a significant decrease in the median time to clinical recovery in the pulmonary irradiation group (3 days vs 12 days, hazard ratio [HR] 2.0, *p* = 0.05). Additionally, they reported that LD-RT improved delirium, radiographs, and biomarkers, with no significant acute toxicity [25]. Moreno-Olmedo et al. have reported preliminary results of the ULTRA-COVID trial (NCT04394182) in 2 patients who received a single 0.8 Gy dose whole lung irradiation through a Tomotherapy. Both patients experienced clinical improvement after LD-RT according to the increase in PaO_2_/FiO_2_ ratio above 300 mm Hg as well as reduced dyspnea, asthenia and bilateral pulmonary infiltrative pattern, visible in the chest CT. In addition, the authors also report a decrease in inflammatory parameters, especially IL‑6 levels [26]. Del Castillo et al. have reported satisfactory results after a single 1 Gy whole lung irradiation for a 64-year-old patient presenting with COVID-19 pneumonia and a rapidly deteriorating respiratory function [27]. Sanmamed et al. (NCT04420390) recently reported on 9 patients with COVID-19 pneumonia who underwent whole lung single 1 Gy fraction with primary endpoint of radiological response. The authors reported a significant improvement in the extension of CT pneumonia as well as in SpO_2_/FIO_2_ at 72 h and 1 week after LD-RT [28]. Finally, Papachristofilou et al. have published the results of the first randomized trial of whole lung LD-RT for the treatment of COVID19 pneumonia (NCT04598581). The authors analyze the results observed in 22 patients randomized to receive a single whole lung dose (1 Gy) or not. Only elderly patients admitted to the ICU who would require intubation and mechanical ventilation were selected. Beyond the technique used for LD-RT, also debatable because it is far from the quality standards required by modern radiotherapy, the results of the study did not demonstrate the benefit of LD-RT in improving the situation of critically ill patients in the ICU, therefore advising against its use. However, as the authors themselves acknowledge, one of the reasons that could justify the lack of benefit lies in the selection of patients, with an extremely critical condition requiring constant mechanical ventilation [29]. Probably, like it has been previously commented, LD-RT should be considered in the initial phases of the exacerbated inflammatory response that accompanies the SARS-CoV‑2 infection, the so-called ‘cytokine storm’, in order to maximize the anti-inflammatory effect of dexamethasone.

One of the main barriers when considering the use of LD-RT for the symptomatic treatment of pneumonia in the context of COVID-19 is the safety of the treatment and the prevention of possible toxicity secondary to radiation therapy. However, none of the previously cited studies have reported complications secondary to radiotherapy. Radiation doses for COVID-19 pneumonia are very low (< 1% of the doses used for cancer radiotherapy), not exceeding tolerance doses for critical organs (heart, thyroid, stomach or kidneys) nor increasing the risk of development of secondary cancers, which also remains extremely low [30–32]. Preclinical studies showed that doses higher than 200 cGy induce a pro-inflammatory effect directly related to late complications with radiotherapy. However, doses below 100 cGy were associated with an anti-inflammatory effect [9]. Thereby, studies with FDG-PET have shown an increased risk of pulmonary toxicity in patients who receive a mean dose to the lung above 2–5 Gy, far from the doses administered for the treatment of COVID-19 pneumonia, as is the case with the hypothetical risk of cardiac toxicity [10]. In a recent study based on a virtual case simulation, a radiation dose ≤ 0.5 Gy provided an acceptable lifetime attributable risks (LAR) estimate (≤ 1%) for radiation-induced cancer (RIC) and cardiovascular risk of exposure-induced death (REID), regardless of sex and age [33]. Nevertheless, the risk must always be taken into consideration. The consequences of not receiving a treatment that is proving its effectiveness should also be evaluated, even more when the target of patients includes those with advanced age and worse clinical situation not candidates for extreme measures, for which the benefit–risk balance certainly appears favorable. The safety of treatments for COVID-19 pneumonia has also been presented in various studies as well as adverse effects has also been associated to other treatments used for the symptomatic relief of patients with COVID-19 pneumonia. Thus, the use of remdesivir has been linked to an appearance of rash, diarrhea, constipation, impaired liver and kidney function and, particularly, cardiotoxicity, which can occasionally be severe [34, 35]. Similarly, the humanized recombinant monoclonal antibody tocilizumab, directed against the IL‑6 receptor, has also been linked to the appearance of complications, sometimes severe, including infections, neutropenia, or alterations in liver enzymes [36].

Our results are in agreement with those reported by other groups. To our knowledge, the present series is the longest of all the experiences published to date and, although it is a nonrandomized trial and the follow-up is still short, observed results suggest a benefit of pulmonary LD-RT for the relief of COVID-19 pneumonia in selected patients. Of the 36 patients initially included in this trial, 8 of them died of COVID-19 disease and 5 of other causes. For 21 of the 25 evaluable patients (84%), the rate of improvement in SpO_2_/FIO_2_ reached 76% after 1 week and respiratory parameters noticeably improved together with a marked reduction in serum inflammation parameters. In those who survived, the CT scan at 1 week after LD-RT showed a significant improvement in the percentage of lung involvement.

Pulmonary LD-RT is an alternative that is worth exploring in the current context of the COVID-19 pandemic and LD-RT should be considered before the inflammatory cascade, which is largely responsible for the symptoms of COVID-19, is completely established. Otherwise, at the moment that the respiratory and clinical condition of patients is at serious risk of fatal deterioration, LD-RT would be unable to reverse it. In addition to the possible efficacy suggested by different studies, other potential advantages of the use of LD-RT in COVID-19 would be that it is not a competitive treatment that would prevent or interfere with the administration of other therapeutic measures, that it is already available in most general hospitals, and that its availability is not subject to stock shortages or market fluctuations.

Not only should the status of COVID-19 pneumonia be an indicator for pulmonary LD-RT, but also should the baseline condition of patients and their cognitive ability to adequately collaborate during and after treatment be taken into account from the beginning. From observed results, we suggest that LD-RT should be considered earlier in the evolution of the disease, in its more initial stages, and for individuals with fewer comorbidities and greater expectations of improvement.

## Conclusions

The use of pulmonary LD-RT, as indicated in this and other ongoing trials, appears to be safe and feasible for patients with COVID-19 pneumonia and deserves to be explored. However, a suitable moment for its use, probably in the earlier stages of the disease, as well as a suitable selection of candidate patients, would be a fundamental requirement if we are to maximize its benefit. Although the present study presented only the results of the first phase of the study including those patients who have received LD-RT, results are encouraging. The analysis of the complete series together with the control group according to the original design of the IPACOVID trial is ongoing and its results are pending publication. Nevertheless, further studies with longer follow-up are necessary to confirm these promising results.

## Supplementary Information

Table A1 Evaluation of CURB-65 score in patients with COVID-19 treated with low-dose radiation therapy (LD-RT) classified by survivors, COVID-19 deaths and deaths from other causes before, at 24 h, 1 week and month after LD-RT

Table A2 Evaluation of functional status and geriatric depression scales in patients with COVID-19 treated with low-dose radiation therapy classified by survivors, COVID-19 deaths and deaths from other causes

Figure A1. Treatment fields of low-dose radiation therapy.

Figure A2. Selected respiratory (A) and biochemical (B) parameters before, 24 h, 1 week and 1 month after radiotherapy treatment in all patients.

Figure A3. Computed tomography (CT) images before (A–E) and 1 week after irradiation (F–J) in one patient with COVID-19 pneumonia treated with low-dose radiation therapy.
