# Supplementary figures and images for: Could pulmonary low-dose radiation therapy be an alternative treatment for patients with COVID-19 pneumonia? Preliminary results of a multicenter SEOR-GICOR nonrandomized prospective trial (IPACOVID trial)
Source: Strahlenther Onkol. 2021 Jul 6;197(11):1010–20. doi: 10.1007/s00066-021-01803-3 (PMC8260020; doi:10.1007/s00066-021-01803-3)

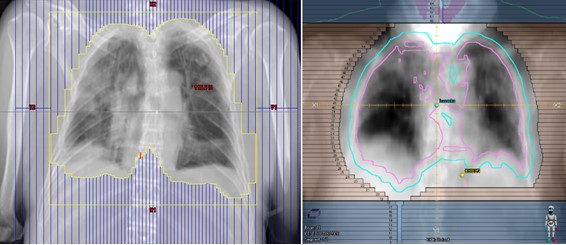

Supplement: Supplementary file 1 — Table A1 Evaluation of CURB-65 score in patients with COVID-19 treated with low-dose radiation therapy (LD-RT) classified by survivors, COVID-19 deaths and deaths from other causes before, at 24 h, 1 week and month after LD-RT [file 66_2021_1803_MOESM1_ESM.jpg]

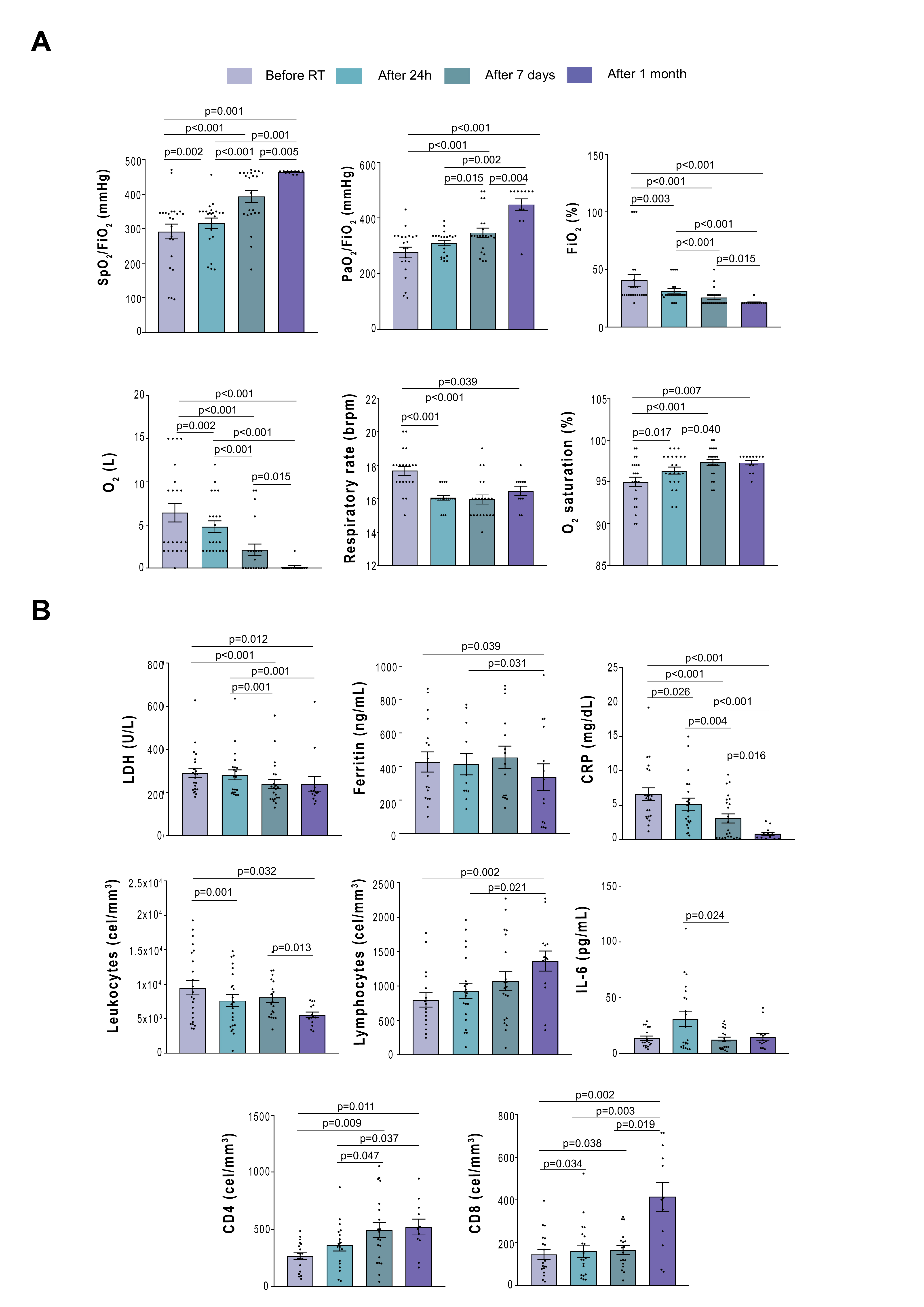

Supplement: Supplementary file 2 — Table A2 Evaluation of functional status and geriatric depression scales in patients with COVID-19 treated with low-dose radiation therapy classified by survivors, COVID-19 deaths and deaths from other causes [file 66_2021_1803_MOESM2_ESM.jpg]

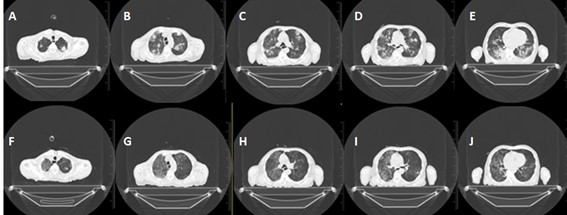

Supplement: Supplementary file 3 — Figure A1. Treatment fields of low-dose radiation therapy. [file 66_2021_1803_MOESM3_ESM.jpg]
