# Supplementary material for: Could pulmonary low-dose radiation therapy be an alternative treatment for patients with COVID-19 pneumonia? Preliminary results of a multicenter SEOR-GICOR nonrandomized prospective trial (IPACOVID trial)
Source: Strahlenther Onkol. 2021 Jul 6;197(11):1010–20. doi: 10.1007/s00066-021-01803-3 (PMC8260020; doi:10.1007/s00066-021-01803-3)
Supplement: Supplementary file 4 — Figure A2. Selected respiratory (A) and biochemical (B) parameters before, 24 h, 1 week and 1 month after radiotherapy treatment in all patients. [file 66_2021_1803_MOESM4_ESM.docx]

**Appendice Table A1**. Evaluation of CURB-65 score in patients with COVID-19 treated with low-dose radiation therapy (LD-RT) classified by survivors, COVID-19 deaths and deaths from other causes before, at 24h, 1 week and month after LD-RT.

|  | **Survivors (n=23)** | | | | **Covid-19 deaths (n=8)** | | **Deaths from other causes (n=5)** | |
| --- | --- | --- | --- | --- | --- | --- | --- | --- |
|  | **Before** | **24h after** | **1 week after** | **1 month after** | **Before** | **24h after** | **Before** | **24h after** |
| **CURB-65 score** |  | | | | | | | |
| 1 point | - | - | 3 (13.6) | 11 (47.8) | - | - | - | - |
| 2 points | 10 (43.5) | 19 (86.4) | 16 (72.7) | 1 (4.3) | - | 1 (16.7)^c^ | - | 3 (60) |
| 3 points | 11 (47.8) | 2 (9.1) | 3 (13.6) | - | 3 (37.5) | 1 (16.7) | 3 (60) | 1 (20) |
| 4 points | 2 (8.7) | 1 (4.5) | - | - | 5 (62.5)^a^ | 4 (66.7)^b^ | 2 (40) | 1 (20) |
