# Supplementary material for: Could pulmonary low-dose radiation therapy be an alternative treatment for patients with COVID-19 pneumonia? Preliminary results of a multicenter SEOR-GICOR nonrandomized prospective trial (IPACOVID trial)
Source: Strahlenther Onkol. 2021 Jul 6;197(11):1010–20. doi: 10.1007/s00066-021-01803-3 (PMC8260020; doi:10.1007/s00066-021-01803-3)
Supplement: Supplementary file 5 — Figure A3. Computed tomography (CT) images before (A–E) and 1 week after irradiation (F–J) in one patient with COVID-19 pneumonia treated with low-dose radiation therapy. [file 66_2021_1803_MOESM5_ESM.docx]

**Appendice Table A2**. Evaluation of functional status and geriatric depression scales in patients with Covid-19 treated with low-dose radiation therapy classified by survivors, Covid-19 deaths and deaths from other causes.

|  | **Survivors**  **(n= 23)** | **Covid-19 deaths**  **(n= 8)** | **Deaths from other causes**  **(n= 5)** |
| --- | --- | --- | --- |
| **Functional status (Barthel scale)** |  |  |  |
| Independent | 5 (21.7) | 1 (12.5) | - |
| Minimally dependent | 10 (43.5) | - | 2 (40) |
| Partially dependent | 3 (13) | 3 (37.5) | 1 (20) |
| Very dependent | 4 (17.4) | 3 (37.5) | 1 (20) |
| Total dependent | 1 (4.3) | 1 (12.5) | 1 (20) |
| **Geriatric Depression Scale (GDS)** |  |  |  |
| No cognitive decline | 16 (69.6) | - | 2 (40) |
| Very mild cognitive decline | 2 (8.7) | 3 (37.5) | 3 (60)^a^ |
| Mild cognitive decline | 3 (13) | 2 (25) | - |
| Moderate cognitive decline | 2 (8.7) | - | - |
| Moderately severe cognitive decline | - | - | - |
| Severe cognitive decline | - | 3 (37.5) | - |
| Very severe cognitive decline | - | - | - |
